# A catalogue of resistance gene homologs and a chromosome‐scale reference sequence support resistance gene mapping in winter wheat

**DOI:** 10.1111/pbi.13843

**Published:** 2022-05-30

**Authors:** Sandip M. Kale, Albert W. Schulthess, Sudharsan Padmarasu, Philipp H. G. Boeven, Johannes Schacht, Axel Himmelbach, Burkhard Steuernagel, Brande B. H. Wulff, Jochen C. Reif, Nils Stein, Martin Mascher

**Affiliations:** ^1^ Leibniz Institute of Plant Genetics and Crop Plant Research (IPK) Gatersleben Seeland Germany; ^2^ Limagrain GmbH Peine‐Rosenthal Germany; ^3^ John Innes Centre Norwich Research Park Norwich UK; ^4^ Center for Desert Agriculture, Biological and Environmental Science and Engineering Division (BESE) King Abdullah University of Science and Technology (KAUST) Thuwal Saudi Arabia; ^5^ Center for Integrated Breeding Research (CiBreed) Georg‐August‐University Göttingen Göttingen Germany; ^6^ German Centre for Integrative Biodiversity Research (iDiv) Halle‐Jena‐Leipzig Leipzig Germany

**Keywords:** wheat, yellow rust, leaf rust, association genetics, resistance gene enrichment sequencing, genome assembly

## Abstract

A resistance gene atlas is an integral component of the breeder’s arsenal in the fight against evolving pathogens. Thanks to high‐throughput sequencing, catalogues of resistance genes can be assembled even in crop species with large and polyploid genomes. Here, we report on capture sequencing and assembly of resistance gene homologs in a diversity panel of 907 winter wheat genotypes comprising *ex situ* genebank accessions and current elite cultivars. In addition, we use accurate long‐read sequencing and chromosome conformation capture sequencing to construct a chromosome‐scale genome sequence assembly of cv. Attraktion, an elite variety representative of European winter wheat. We illustrate the value of our resource for breeders and geneticists by (i) comparing the resistance gene complements in plant genetic resources and elite varieties and (ii) conducting genome‐wide associations scans (GWAS) for the fungal diseases yellow rust and leaf rust using reference‐based and reference‐free GWAS approaches. The gene content under GWAS peaks was scrutinized in the assembly of cv. Attraktion.

## Introduction

Maintaining plant health in the face of evolving pathogen populations is a perennial goal of breeders. Key to this endeavour is the discovery and deployment of disease resistance (R) genes. Hafeez *et al*. (2021) put forward the concept of an R gene atlas and illustrated its potential for crop improvement in one of our most widely grown crops, wheat. An important component of populating the wheat R gene atlas is genotyping diversity panels, or more broadly, knowledge of as large a fraction of the resistance gene complement of as many genotypes as possible. One approach to this aim, resistance gene enrichment sequencing [RenSeq, Jupe *et al*. (2013)], was developed with large‐crop genomes in mind. To reduce the genomic complexity of sequencing libraries, and hence the required sequence effort, capture probes are designed to target R gene homologs from the nucleotide‐binding and leucine‐rich repeat (NB‐LRR) family, or more generally, the family of NB‐LRR‐related genes (NLRs, Ting *et al*. (2008)). In its original implementation, RenSeq was combined with short‐read sequencing on the Illumina platform (Jupe *et al*., 2013). A combination of RenSeq with long‐read sequencing has been used to assemble the full complement of R genes in the model plant *Arabidopsis thaliana* and analyse their evolutionary dynamics (Van de Weyer *et al*., 2019).

RenSeq data for diversity panels in combination with matching phenotype data have been used for genome‐wide associations scans (GWAS) to find genetic markers associated with disease resistance (Arora *et al*., 2019). In the best case, this method, termed AgRenSeq, can zoom in on individual candidate genes. However, the limits of association mapping such as population structure (Yu *et al*., 2006) and sensitivity to the genetic architecture of the trait under study (Lopez‐Arboleda *et al*., 2021) also apply to AgRenSeq. Recently, Gaurav *et al*. (2021) reported the use of whole‐genome shotgun sequencing for association mapping of disease resistance in the wheat diploid progenitor *Aegilops tauschii*. An advantage of WGS over RenSeq is its ability to access also non‐NLR resistance genes; a potential drawback is the inability to assemble full‐length genes from low to medium‐coverage (3×–10×) short‐read data.

Independent of the choice of sequencing strategy, a potential impediment to GWAS and a crucial aspect of R gene evolution is structural variation (SV). R genes are subject to ubiquitous presence‐absence and copy‐number variations (Michelmore and Meyers, 1998; Van de Weyer *et al*., 2019). Reference‐free GWAS approaches have shown that the presence of peaks can be influenced by the choice of the reference sequence (Voichek and Weigel, 2020). In principle, the best resource for studying intra‐species NLR diversity are high‐quality genome assemblies for a representative diversity panel comprising hundreds of accessions, i.e. a pan‐genome. Constructing pan‐genome infrastructures for all major crops has recently turned from a moon shot into a realistic mid‐term research goal (Della Coletta *et al*., 2021). But the wheat pan‐genome is not there yet: chromosome‐scale reference genome sequences for ten wheat varieties, most of them recent elite cultivars, have recently been released (Walkowiak *et al*., 2020), but this small panel is not comprehensive enough to underpin a species‐wide resistance gene inventory.

In the present manuscript, we report on a contribution to the wheat R gene atlas. We constructed an R gene inventory for a diversity panel of winter wheat, the predominant type of wheat in Europe. A chromosome‐scale reference genome sequence was constructed for one representative winter wheat cultivar. To illustrate the value of this resource for the wheat genetics and breeding community, we (i) compare patterns of R gene diversity between plant genetic resources (PGR) and elite cultivars; (ii) conduct GWAS for the fungal diseases yellow rust and leaf rust; and (iii) analyse structural variants in close proximity to significantly associated markers.

## Results

### R gene capture in a winter wheat diversity panel

**Figure 1:**
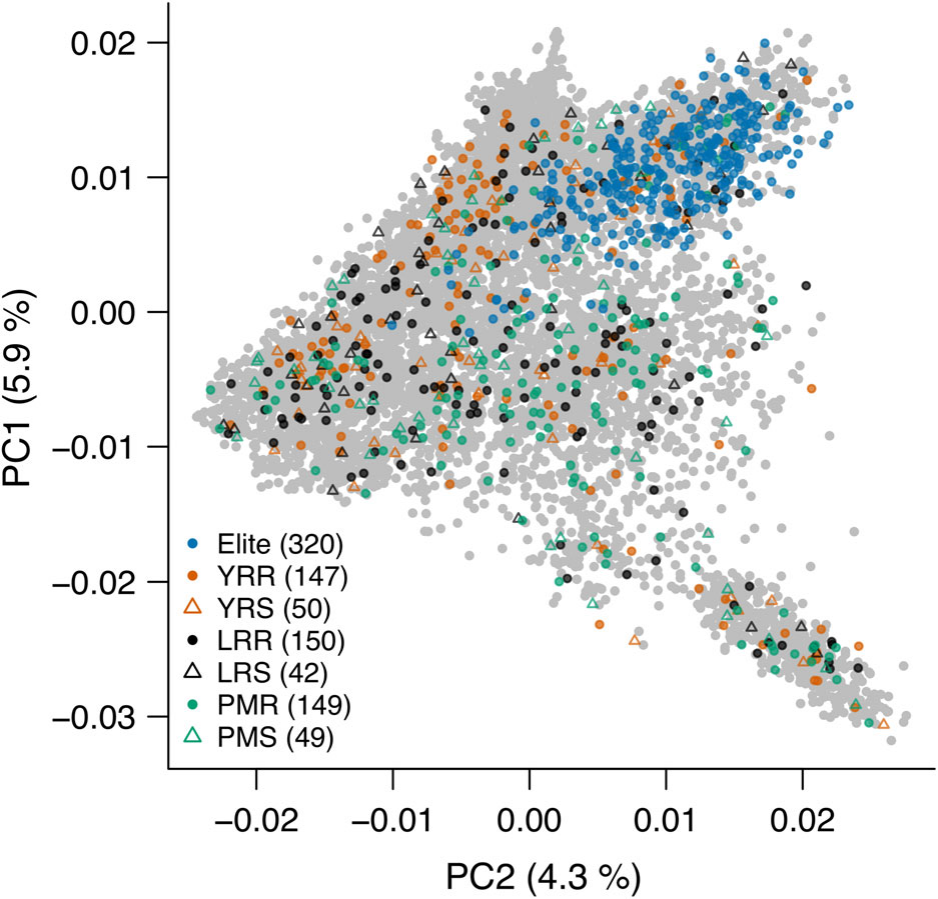
Genetic diversity of genotypes selected for RenSeq. The 907 accessions selected for the RenSeq analysis were projected onto the molecular diversity space of the winter wheat collection of the IPK genebank portrayed by the first two principal components (PCs) from a PC analysis on genome‐wide SNP markers (Schulthess *et al*., 2021). Among RenSeq characterizations, 192 European elite cultivars and 128 German elite breeding lines represent the diversity already handled by European breeding. The remaining fraction is composed of 587 plant genetic resources (PGRs) samples from the IPK genebank, which was enriched for disease‐resistant genotypes with minimized population structure (Schulthess *et al*., 2021). According to selection, PGRs are classified as yellow rust‐resistant [YRR] or susceptible [YRS], leaf rust‐resistant [LRR] or susceptible [LRS] and powdery mildew‐resistant [PMR] or susceptible [PMS].

We conducted RenSeq for a panel of 907 winter wheat genotypes (Figure 1, Table S1) and the reference genotypes Chinese Spring (The International Wheat Genome Sequencing Consortium (IWGSC), 2018) and Julius (Walkowiak *et al*., 2020). Of these, 779 are part of a previously described core set enriched for disease‐resistant genotypes (Schulthess *et al*., 2021) comprising 587 PGRs and 192 European elite cultivars. The remaining 128 genotypes are recent German elite breeding lines. We used the Triticeae RenSeq Baits V3 (Tv3) probe set comprising 217 827 oligonucleotide baits (Zhang *et al*., 2021). Alignment of this bait set to the Chinese Spring reference genome (RefSeq v1.0, The International Wheat Genome Sequencing Consortium (IWGSC) (2018)) indicated that 18 Mb of annotated NBS‐LRR gene sequence are targeted. On average, sequences originating from the predicted target were enriched 220‐fold.

**Figure 2:**
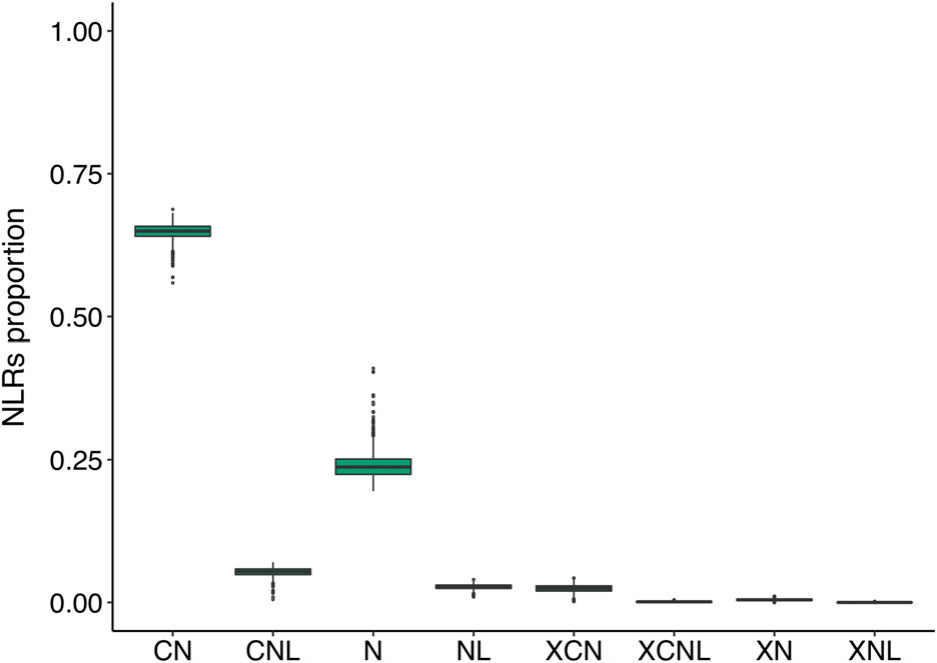
Proportion of NLRs of different classes in individual RenSeq assemblies. CN—Coiled‐coil NBS; N—NBS; CNL—Coiled‐coil NBS‐LRR; NL—NBS‐LRR; XCN—integrated domain (ID)‐Coiled‐coil NBS; XCNL—ID‐Coiled‐coil NBS‐LRR; XNL—ID‐NBS‐LRR.

RenSeq reads of individual genotypes were assembled *de novo*, yielding 67 731 to 4 583 579 contigs per accession (Table S2). Of these, 417 to 2304 per accession (mean: 1690) contained full‐length NLRs. Coiled‐coil NLRs were the most abundant class of NLRs (Figure 2). Almost all (1923/1937) NLRs assembled from the Chinese Spring RenSeq data were aligned to RefSeq v1.0. A total of 1486 (77%) Chinese Spring *de novo* assembled NLRs overlapped with RefSeq v1.0 gene models, indicating the absence of resistance gene homologs in the reference annotation, possibly because of lack of expression or pseudogenization. In other genotypes, on average 77% of assembled NLRs were mapped to RefSeq v1.0, consistent with pervasive presence‐absence variation (PAV) in resistance genes.

### Diversity of NLRs in winter wheat genepools

To understand the extent of PAV in NLRs in our panel, we performed similarity‐based clustering of the assembled NLR from all genotypes. A total of 1 469 694 (96%) NLRs were clustered in 39 073 orthogroups, the remainder were singletons without close matches to other NLRs. Fewer than 1% of orthogroups contained two or more NLRs from the same accession, pointing to a potential collapse of highly similar, recently duplicated NLRs. Most (>85%) of orthogroups had NLRs from at least 20 different accessions. However, very few orthogroups (472, 1.21%) had members from more than 500 accessions (Figure S1). This is at odds with patterns of NLR diversity in the model plant *Arabidopsis thaliana* (Van de Weyer *et al*., 2019), where the “core‐NLRome” comprising genes present in almost all genotypes is substantial. The likely explanation is random sequence dropout due to competition between capture probes and/or low sequencing depth. For example, at a 1% dropout rate (i.e. a 99% chance of being captured and sequenced at sufficient depth), a gene present in 900 genotypes has a negligible chance (0.99^900^ = 0.01%) of being present in all their assemblies.

**Figure 3:**
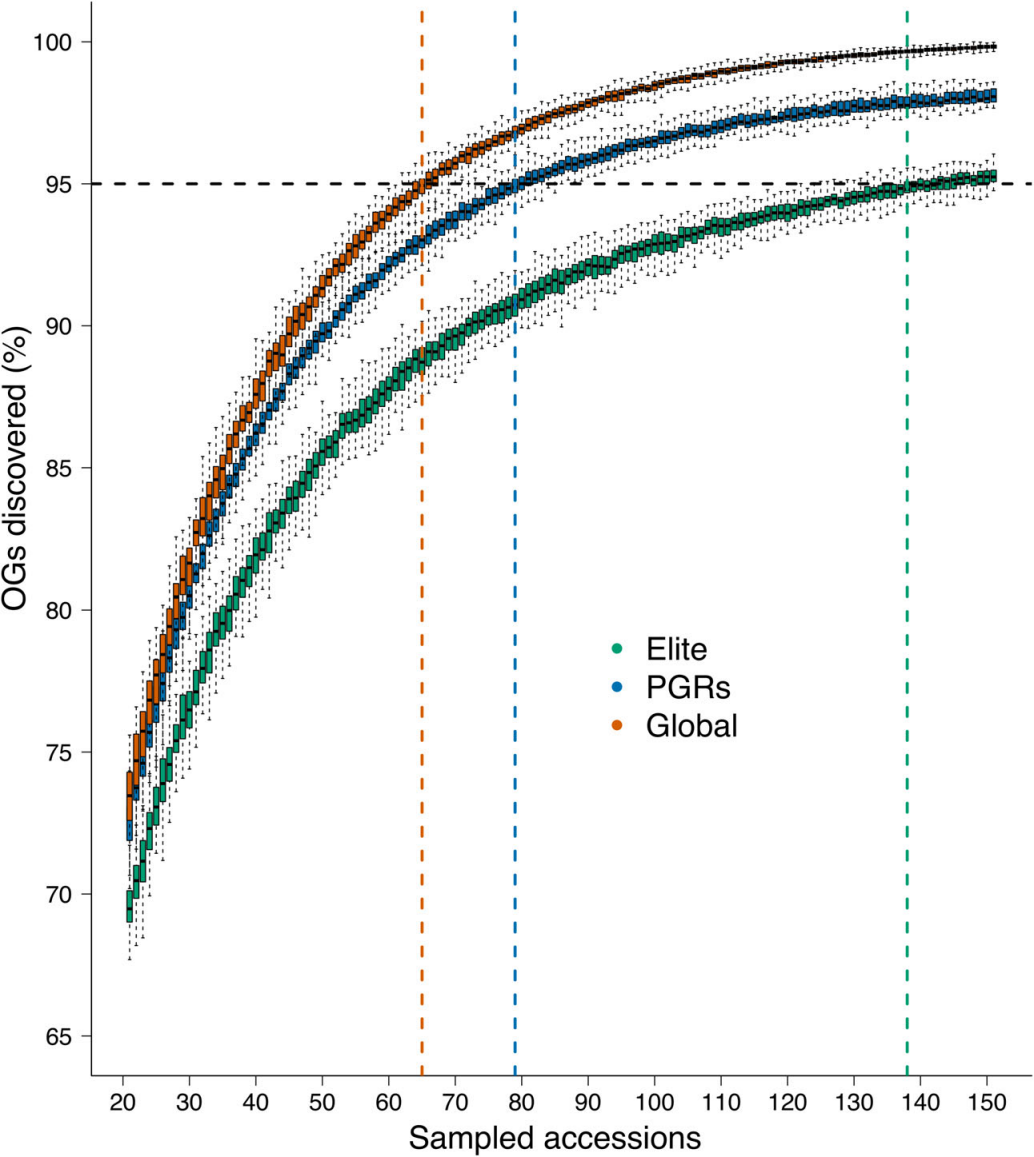
Saturation analysis. Fraction of NLR orthogroups recovered from randomly drawn subsets of genotypes. Subsets were selected from the entire population, and elite varieties and PGRs. Sampling was repeated 100 times for subsets of increasing size. Colored vertical lines indicate the number of accessions required to achieve 95% representation of the NLR universe.

**Figure 4:**
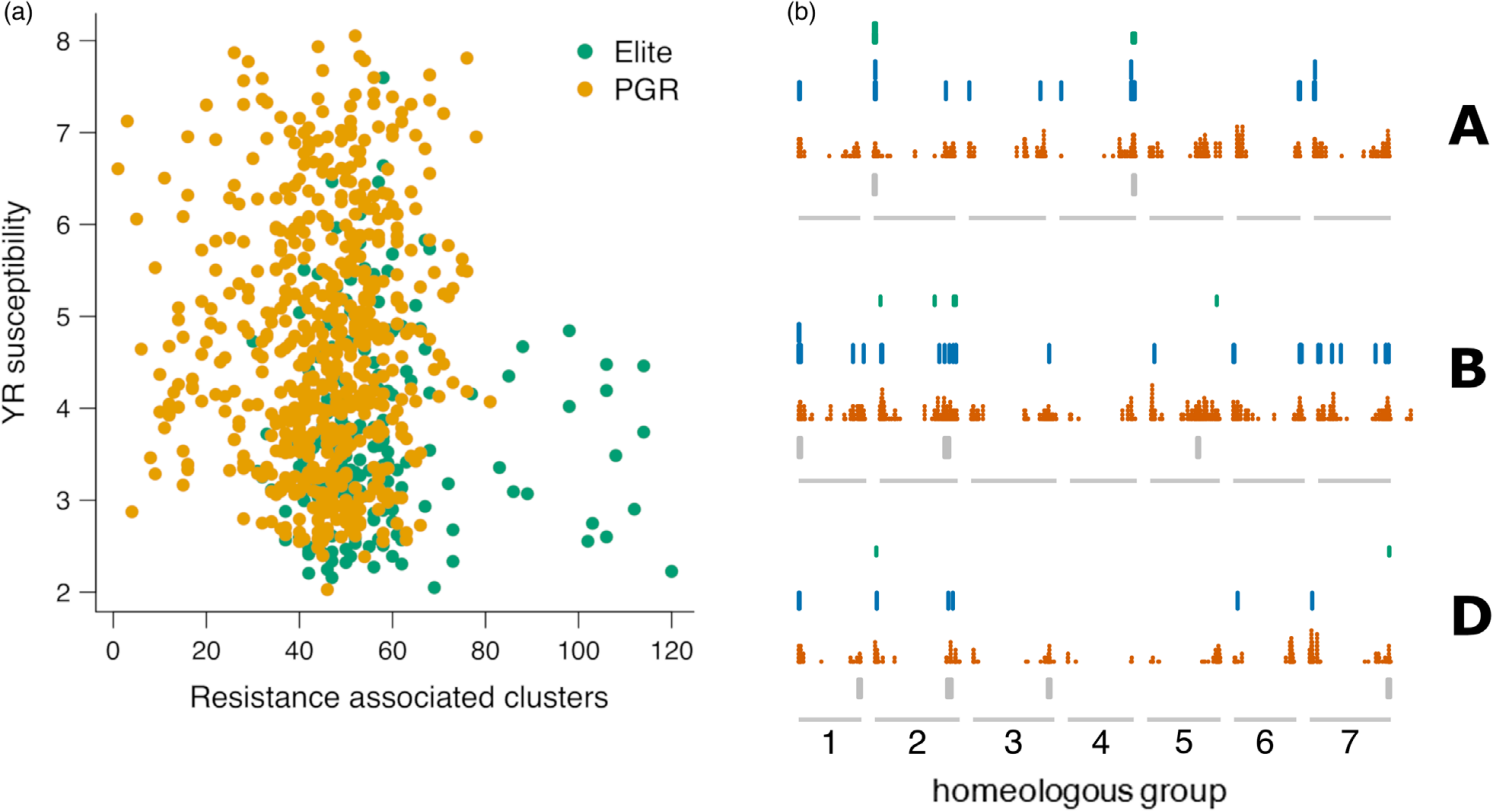
Enrichment of resistance‐associated NLR clusters in elite lines. (a) The number of resistance‐associated clusters from an accession is plotted against its yellow rust susceptibility score. Elite lines with many resistance‐associated clusters were less susceptible to yellow rust. (b) Genomic distribution of elite‐specific orthogroups (OGs, green), PGR‐specific OGs (blue) and OGs present in both elite lines and PGRs (orange) in the three subgenomes of hexaploid wheat (A, B, D). The grey boxes mark the positions of alien introgressions.

A saturation analysis indicated that a near‐complete set of NLR orthogroups assembled in the whole panel can be captured with a rather small number of accessions: 95% of orthogroups were captured with only 65 genotypes selected at random from the universe of 907 accessions (Figure 3). Because of random dropout, these figures are likely overestimates, i.e. an even smaller panel may suffice to reach the 95% threshold. Still, the analysis of orthogroups allows for the comparison of relative diversity between gene pools. When considering PGR and elite accessions separately, near‐saturation can be achieved with 79 and 138 accessions, respectively, indicating that, not unexpectedly, NLR diversity is higher in PGRs. However, elite lines were more resistant against yellow rust compared with PGRs and contained a higher number of NLRs that preferentially occur in resistant genotypes, supporting the notion that the breeder’s efforts to stack resistance genes have been successful (Figure 4a). A potential caveat, though, is that NLRs private to elite varieties were localized to regions previously reported as harbouring alien introgressions. By contrast, PGR‐specific NLRs tended to be distributed more uniformly across the chromosomes (Figure 4b). This is consistent with the notion that most NLRs occurring in highly resistant elite varieties may not confer resistance on their own but have only hitchhiked along one or a few functional resistance genes targeted by breeders.

### A chromosome‐scale assembly of cv. Attraktion

Several recent studies suggest that the choice of reference genome impacts the contextualization or even the very presence of GWAS peaks (Arora *et al*., 2019; Voichek and Weigel, 2020). It is likely that a better reference genome than the assembly of Chinese Spring—indeed a spring‐sown landrace from China (Sears and Miller, 1985)—can be selected for mapping resistance genes in winter types. We chose cv. Attraktion because our prior analysis of shallow‐coverage whole‐genome shotgun data had shown that this cultivar carries large alien introgressions, some of them co‐incident with GWAS peaks for resistance to yellow and leaf rust (Schulthess *et al*., 2021).

We sequenced the Attraktion genome to 22‐fold coverage with HiFi reads with an average length of 17.8 kb of circular consensus reads. In addition, chromosome conformation capture sequencing (Hi‐C) was performed, resulting in 994 million read pairs. Genome assembly following a previously described approach (Mascher *et al*., 2021; Sato *et al*., 2021) combining primary contig assembly with Hifiasm (Cheng *et al*., 2021) and pseudomolecule construction with the TRITEX pipeline (Monat *et al*., 2019) yielded a set of 1553 contigs (14.25 Gb) assigned to chromosomal locations. A further 3442 contigs (434 Mb) remained unplaced. A BUSCO analysis (Simao *et al*., 2015) indicated that 98.2% of conserved single‐copy genes were present in the assembly (Table 1). The inspection of Hi‐C contact matrices and alignment to the Chinese Spring RefSeq v2.1 (Zhu *et al*., 2021) supported the structural integrity of the pseudomolecules (Figures S2 and S3).

**Table 1:** Statistics of the genome sequence assembly of cv. Attraktion

Assembly size	14.7 Gb
Number of contigs	4953
Contig N50	17.3 Mb
Contig N90	4.1 Mb
Pseudomolecule size	14.3 Gb
Number of contigs in pseudomolecules	1553
Complete BUSCOs	1584 (98.2%)

**Figure 5:**
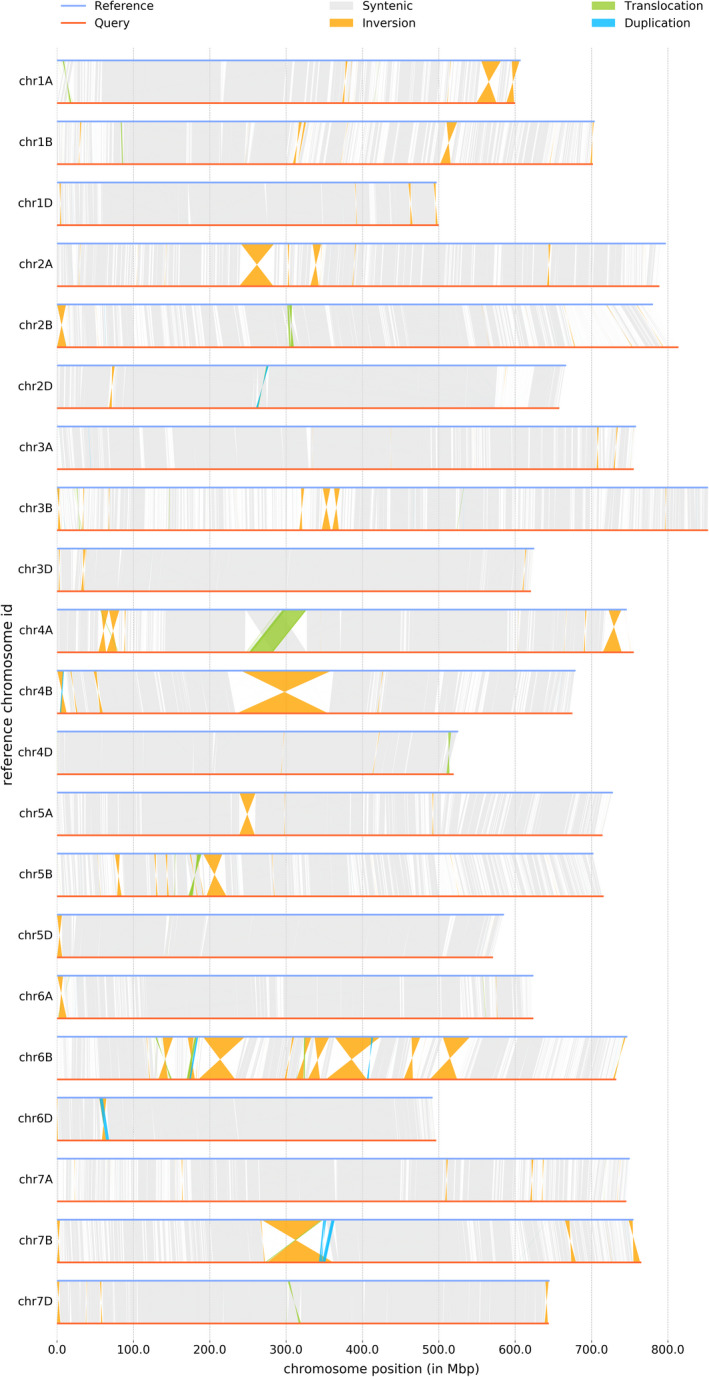
Structural variants detected by Synteny and Rearrangement Identifier (SyRI, Goel *et al*., 2019) between the genome assemblies of cv. Attraktion (reference) and Chinese Spring RefSeq V2.1 (query).

Regions of high divergence between Attraktion and Chinese Spring indicative of the presence of alien introgressions were found on four chromosomes: 4A, 2B, 5B and 2D (Figures 5 and S4). The introgressions on chromosomes 4A and 2B had been characterized using genomic data of wild relatives and pedigree information (Przewieslik‐Allen *et al*., 2021; Walkowiak *et al*., 2020). Interestingly, a 55 Mb introgression on the long arm of chromosome 2B in Attraktion overlapped with a much larger 427 Mb introgression from *T*. *timopheevii* in LongReach Lancer (Walkowiak *et al*., 2020). Attraktion and Lancer have the same haplotype in the overlapping region, pointing to shared ancestry (Figure S4). Most likely, breeders had decreased the size of this introgression in Attraktion in an attempt to reduce linkage drag.

### Different GWAS approaches identify a yellow rust resistance locus on chromosome 6A

To illustrate the value of our resource for genetic mapping of disease resistance, we conducted GWAS for yellow rust (*Puccinia striiformis* f. sp. *tritici*) resistance in our panel. The degree of yellow rust infection was scored in multi‐environment field trials, relying on natural and artificial infection. Details are described elsewhere (Schulthess *et al*., 2021). We followed three different approaches to obtain matrices of bi‐allelic markers for use in GWAS. First, we aligned RenSeq reads to a reference genome sequence assembly, called single‐nucleotide polymorphisms (SNPs) and used genotype calls at SNP sites as markers. This is the most commonly applied approach for marker discovery, which, however, can capture structural variants only if they are in close linkage disequilibrium (LD) with SNPs. Second, we conducted kmerGWAS (Voichek and Weigel, 2020), which queries the presence‐absence state of short oligonucleotides of a fixed length (*k*‐mers, *k* = 31) as proxies for structural variants. Third, we used SNP sites discovered from the alignment to the reference assembly, but instead of allelic status, we used presence‐absence states of genotype calls as markers, similar to what Gabur *et al*. (2018) did with SNP chip data of rapeseed. We refer to this method as paGWAS. Two different reference genome sequences, Chinese Spring RefSeq v2.1 and our Attraktion assembly were used to position markers. Note that kmerGWAS is a reference‐free approach; associated *k*‐mers were aligned *post hoc* to the genome assemblies to place them.

**Figure 6:**
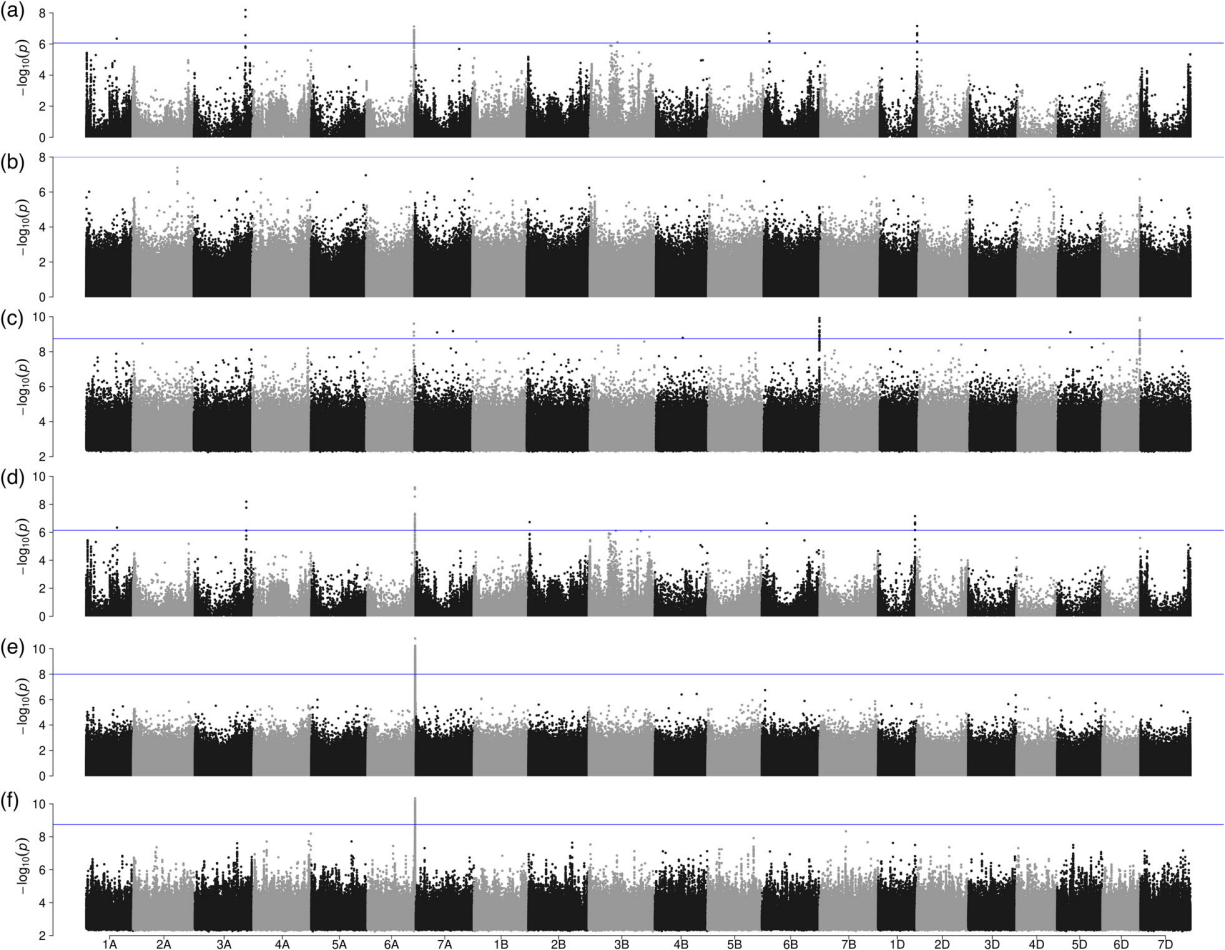
Association scans for yellow rust resistance using different marker systems. Manhattan plots showing GWAS results for yellow rust resistance based on (a) SNPs identified relative to Chinese Spring RefSeq V2.1, (b) presence‐absence GWAS using SNPs identified relative to Chinese Spring RefSeq V2.1 and scored as presence‐absence markers. (c) *k*‐mers mapped against Chinese Spring RefSeq V2.1. Panels (d), (e) and (f) show the results of SNP‐based, presence‐absence and *k*‐mer‐based GWAS when the reference sequence of cv. Attraktion was used for SNP identification or *k*‐mer mapping. The blue horizontal lines indicate the threshold above which associations are statistically significant.

Manhattan plots for all three methods and the two references are shown in Figure 6. The most prominent feature is a peak on the long arm of chromosome 6A, for which significantly associated markers were reported in GWAS scans. However, it is less prominent in paGWAS and kmerGWAS against the Chinese Spring reference, possibly reflecting the absence of the resistant haplotype in that genotype. SNP GWAS against Chinese Spring did result in a pronounced peak, likely because of SNPs in linkage disequilibrium with the causal variant. Further peaks were observed on other chromosomes but were not common between all methods.

**Figure 7:**
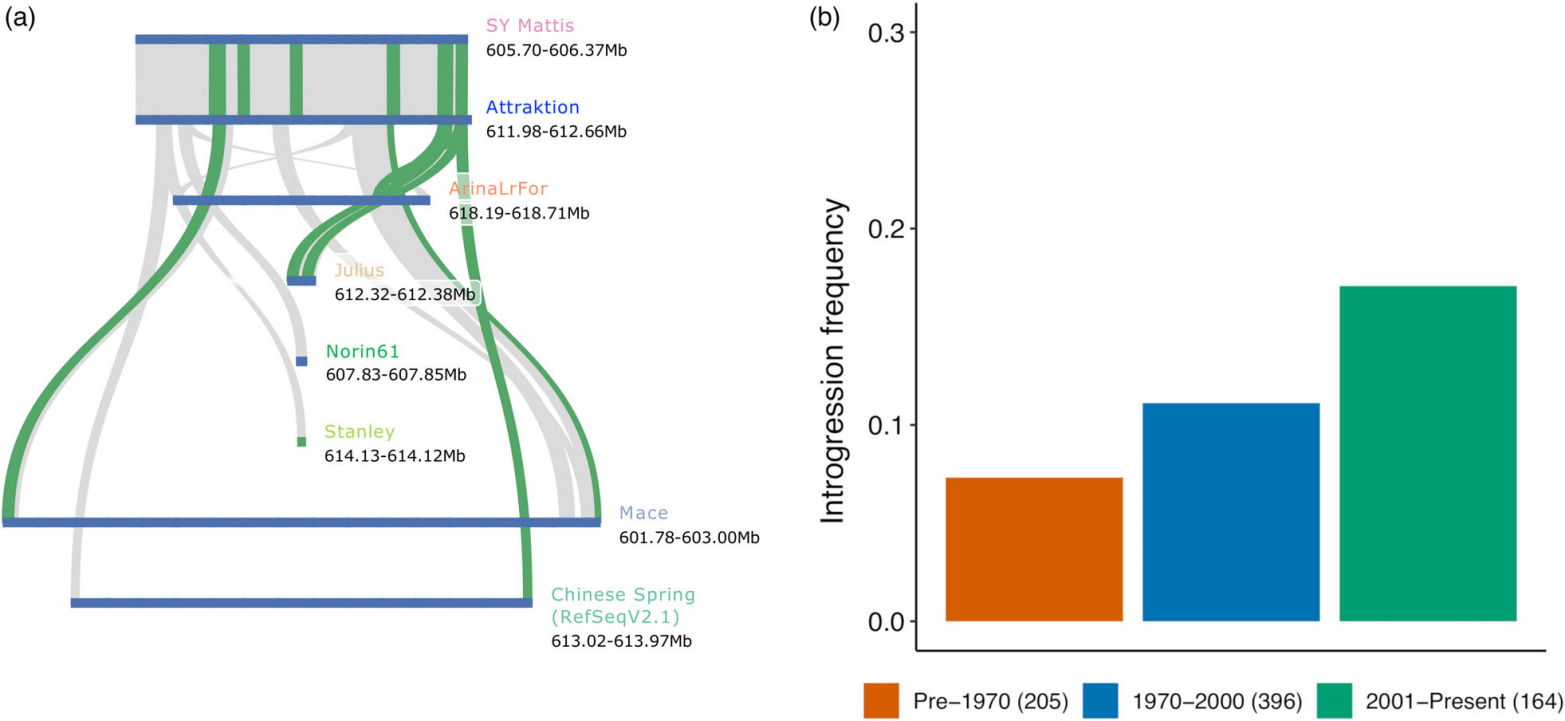
Tracing the history of a novel yellow rust resistance locus on chromosome 6A. (a) Gene‐based collinearity analysis of the yellow rust resistance locus identified on chromosome 6A in the Attraktion assembly with reference assemblies from the wheat pan‐genome (Walkowiak *et al*., 2020). (b) Frequency of resistant haplotypes in accessions from different time period. The numbers in parentheses indicate size of each group.

To the best of our knowledge, a resistance gene against yellow rust has not been reported on chromosome 6A in the region pinpointed by our GWAS. We scrutinized the region under the peak in the genome assembly of cv. Attraktion, which scored highly in our resistance trials and carried the resistant haplotype at the 6A peak. The significantly associated markers spanned an interval of 679 kb (611 981 803 to 612 660 230 bp) in the Attraktion genome (Figure 7a), containing 121 gene models annotated *ab initio* (Stanke *et al*., 2006), many of which are actually derived from transposable elements. Seven of these genes were NLRs. One of them, spanning a 4.9 kb at around sequence coordinate 612.5 Mb on the 6A pseudomolecule of Attraktion, was identical to the representative contig of the orthogroup “cluster49707” identified from the RenSeq *de novo* assemblies. This representative contig harboured 1147 (46.9%) out of 2446 significantly associated 31‐mers, supporting a strong genetic association of that contig with YR resistance. Among the 10 genome sequence assemblies reported by Walkowiak *et al*. (2020), that of SY Mattis had the same haplotype as Attraktion (Figure 7a). The other nine genomes and the Chinese Spring reference lacked several genes present in the Attraktion haplotype, including cluster49707. We could not ascertain the resistance of SY Mattis as we had not included it in our field trials and did not identify a YR isolate that might be recognized by resistance‐conferring NLRs, so that resistance scoring in the laboratory was not possible as well.

Interestingly, no significant marker‐trait associations were detected in the peak region when only PGRs were included in the association scan (Figure S5). The high synteny between Chinese Spring and Attraktion in the vicinity of the peak rules out the presence of an alien introgression. The resistant haplotype was segregated at low frequency (8%) in landraces and old varieties before 1970. Recent shifts in European pathogen populations (Hovmøller *et al*., 2016) may have favoured its rise in European winter wheats (Figure 7b). Future work should focus on the identification and validation of the causal gene conferring yellow rust resistance and on singling out the yellow rust isolates that it recognizes.

### GWAS for leaf rust detects known and novel loci

**Figure 8:**
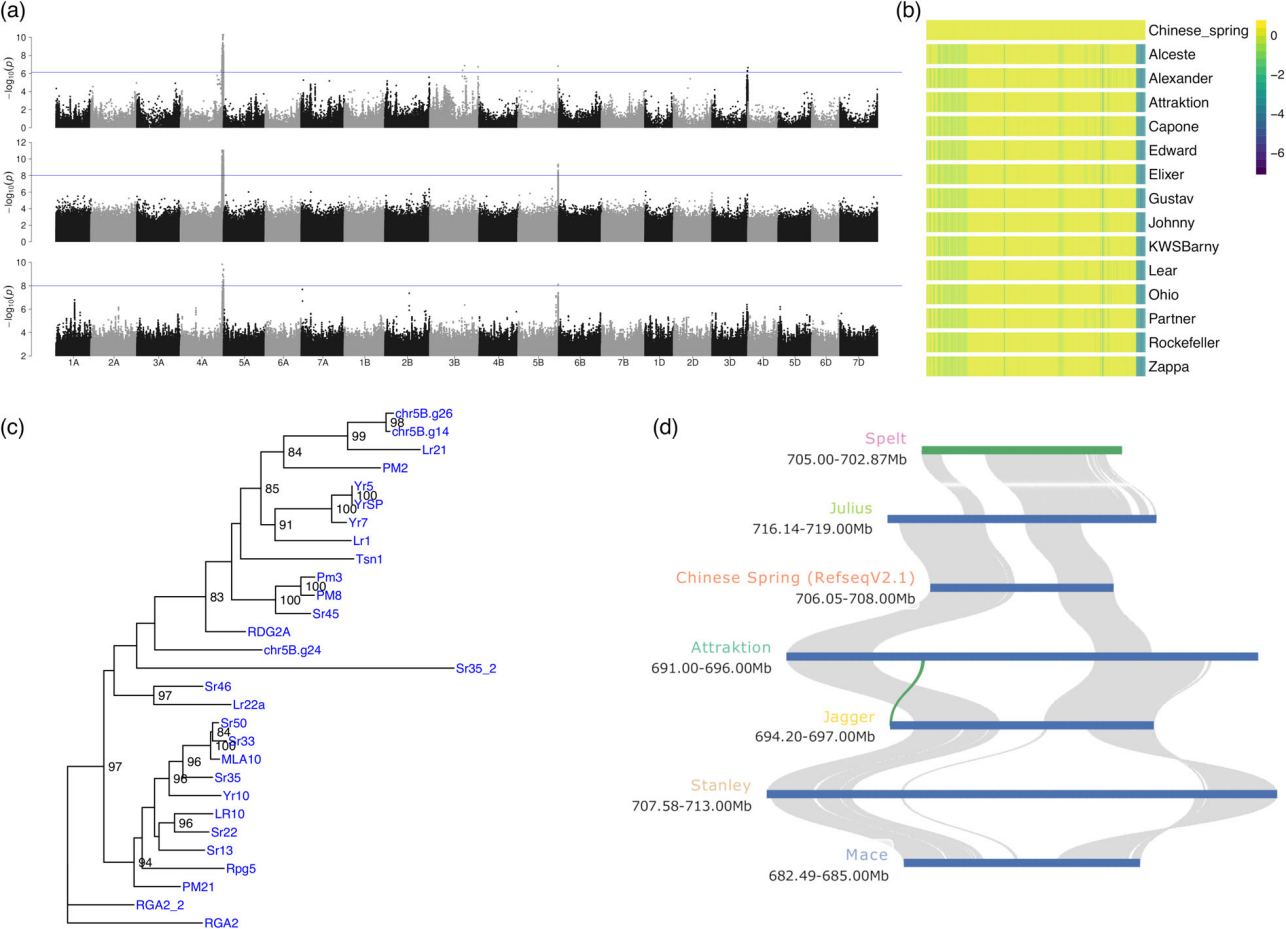
Identification of a leaf rust resistance locus. (a) Manhattan plots showing GWAS results for leaf rust resistance based on SNPs (top row), SNPs scored as presence‐absence markers (middle row) and *k*‐mer markers. SNPs and *k*‐mers were anchored to the reference sequence assembly of cv. Attraktion. Two regions at the distal ends of the long arms of chromosomes 4A and 5B were associated with leaf rust resistance. (b) Normalized read depth in 500 kb bins along chromosome 4A of Chinese Spring RefSeq V1.0 for representative elite varieties. (c) Phylogenetic tree constructed with NBS‐LRR genes from the 5B locus together with cloned resistance genes of wheat. Two genes from the locus are highly homologous to *Lr21*. (d) Gene‐based collinearity analysis of the Attraktion haplotype at the 5B locus with assemblies from the wheat pan‐genome (Walkowiak *et al*., 2020), none of which carry the Attraktion haplotype.

The second trait for which we did GWAS is leaf rust (*Puccinia triticina* f. sp. *tritici*) resistance. The degree of natural infection with leaf rust was scored under field conditions (Tables S3 and S4). SNP, paGWAS and kmerGWAS gave partially overlapping results (Figures 8a and S6). Common to all approaches was a peak towards the distal end of the long arm of chromosome 4A. This association had been reported before by Liu *et al*. (2020a), who analysed 133 genotypes and 1574 of their hybrid offspring by exome sequencing. The GWAS peak was co‐located with a 26 Mb region of high sequence divergence between Chinese Spring and Attraktion (Figure 5), indicative of the presence of an alien introgression. Attraktion is identical by descent to cv. Robigus in a region on chromosome 4A (Figure S4), which Przewieslik‐Allen *et al*. (2021) had shown by the SNP genotyping of wild relatives and perusal of pedigrees to trace back to *T*. *dicoccoides*. Because of suppressed recombination, the introgression is inherited as one large linkage block (Figure 8b).

Significant associations on the long arm of chromosome 5B were detected by multiple GWAS approaches. Cultivar Attraktion had high resistance scores and had a haplotype associated with resistance in the peak region, which extended from about 692.4 to 694.1 Mb in the Attraktion genome assembly, spanning 235 gene models. Of these, thirteen were NLRs. Significantly associated *k*‐mers mapped to 15 orthogroups of NLRs *de novo* assembled from our RenSeq data, which corresponded to three gene models in the Attraktion assembly. One of them was highly similar to the cloned *Lr21* resistance gene (Figure 8c) (Huang *et al*., 2003). Interestingly, none of the wheat pan‐genome assemblies (Figure 8d) (Walkowiak *et al*., 2020) harboured this gene, illustrating the need for bespoke genome sequence assemblies to capture the gene content of resistance gene clusters. Further work is needed to prove the causal link between resistance to leaf rust and the presence of the *Lr21*‐related gene or other NLRs under the GWAS peak.

## Discussion

We have reported RenSeq assemblies as a component of the burgeoning wheat R gene atlas. Due to shortcomings of our short‐read capture sequencing approach, we were unable to construct a comprehensive NLRome as was done with long‐reads in *A. thaliana* (Van de Weyer *et al*., 2019). However, our dataset did reveal a contrasting repertoire of R gene homologs in elite varieties and PGRs, documenting breeders’ efforts at enhancing genetic resistance by selecting haplotypes bearing R genes.

The assembly of one recent elite cultivar, Attraktion, proved instrumental in the analysis of the gene content surrounding two GWAS peaks on chromosomes 6A and 5B for yellow rust and leaf rust, respectively. But no single genotype can capture all resistance genes and sequence assemblies of other genotypes will be required to zoom in on candidate genes against other diseases or other isolates of yellow rust. Fortunately, the cost for wheat whole‐genome assembly has decreased substantially in recent years. The Attraktion assembly was completed within three months after the selection of that genotype and cost approximately EUR 40 000. This shows that whole‐genome assembly still entails large expenses, which, however, may constitute a worthwhile investment if candidate genes cannot be pinned down by cheaper alternatives. As long as capture and selective sequencing of large (>500 kb) genomic regions have not become routine (López‐Girona *et al*., 2020), whole‐genome assembly is a viable alternative even if only a single locus is of interest.

Reference genome sequences of diversity panels large enough for GWAS (i.e. 100–1000 genotypes) would render both RenSeq and WGS superfluous: the comprehensiveness and context afforded by genome assembly cannot be matched by short‐read approaches. However, the large size of the wheat genome (15 Gb) makes the assembly of hundreds or thousands of genotypes cost‐prohibitive at the time of writing. Consequently, the resources reported in this article will retain their usefulness in the medium term.

The three different GWAS approaches we took, SNP GWAS, paGWAS and kmerGWAS, were only partially concordant, highlighting the potential benefits that may be reaped from an integration of the GWAS and pan‐genomics toolkits. Computational frameworks to construct and analyse pan‐genome graphs are under active development (Hickey *et al*., 2020; Li *et al*., 2020). Reduced‐representation approaches focussing on the single‐copy or repeat‐depleted part of the genome have been applied in soybean (Liu *et al*., 2020b) and barley (Jayakodi *et al*., 2020). It is unlikely, however, that a single‐copy sequence can represent copy‐number variation in rapidly evolving resistance genes. Future algorithmic work should focus on the graph‐based representation of pan‐genomes for complex plant genomics, graph‐based read mapping and GWAS with multi‐allelic structural variants captured in pan‐genome graphs.

## Experimental procedures

### DNA extraction, library preparation and sequencing for RenSeq

A total of 779 genotypes from a trait‐customized core collection of winter wheat (Schulthess *et al*., 2021) along with 128 advanced elite lines were used for resistance gene enrichment sequencing (RenSeq). DNA was extracted from a single leaf of about 10 cm length harvested from a 10‐day‐old seedling using the DNeasy 96 Plant Kit (QIAGEN, Hilden, Germany) as per the manufacturer's instructions. DNA quality and quantity were determined using a 0.8% agarose gel and Qubit fluorometer (Life Technologies, Carlsbad, CA). The RenSeq libraries were prepared using the protocol of Steuernagel *et al*. (2017) with minor technical modifications. Briefly, 1 µg DNA from each genotype was fragmented to ~500 bp size using the Covaris S2 (Covaris, MA). The fragmented DNA was purified using 0.6X AMPure® XP beads (Beckman Coulter, IN) according to the manufacturer’s instructions. Paired‐end libraries for Illumina sequencing were constructed using NEBNext® Ultra^TM^ II DNA Library Prep Kit for Illumina® (New England Biolabs Inc, Ipswich, MA) as per the manufacturer’s instructions, except AMPure® XP beads were used for all the purification and size selection steps. For PCR amplification, 10 µl of adapter‐ligated DNA from each genotype was used along with 25 µl 2× KAPA HiFi HotStart ReadyMix (Kapa Biosystems, Wilmington, MA), 1 µl Index and Universal PCR Primer and 13 µl water. The library from each genotype was indexed using Unique Dual Index Primer Pairs (NEBNext Multiplex Oligos for Illumina, Inc., San Diego, CA, USA) in order to perform multiplexed sequencing.

The enrichment of NBS‐LRR DNA fragments was achieved through hybridization of PCR amplified genomic DNA libraries prepared above with 200K Triticeae NLR bait libraries (Tv3, Zhang *et al*. (2021)) available at https://github.com/steuernb/MutantHunter/blob/master/Triticea_RenSeq_Baits_V3.fasta.gz. The libraries were quantified using Qubit fluorometer (Life Technologies, Carlsbad, CA), and the average fragment size was determined using the 4200 Tape Station (Agilent Technologies, Santa Clara, CA). The libraries from eight genotypes were pooled in an equimolar manner and hybridized with the bait library (myBaits‐11; Arbor Biosciences, Ann Arbor, MI). The hybridization reaction was carried out at 65°C for 18 h and the hybridized fragments were captured using MyOne Streptavidin C1 magnetic beads (ThermoFisher Scientific). The hybridization and capture of NBS‐LRR fragments were performed according to MYbaits v4.0 protocol. Finally, PCR amplification of captured fragments was carried out using 2× KAPA HiFi HotStart ReadyMix and standard Illumina P5 and P7 primers. Twelve capture libraries (96 genotypes) were pooled in equimolar amounts, quantified using qPCR and sequenced (paired‐end, 2 × 250 cycles) on the NovaSeq 6000 (Illumina).

### DNA extraction, library preparation and sequencing for PacBio HiFi sequencing

High molecular weight (HMW) DNA for PacBio circular consensus sequencing (CCS) was prepared from 100 one‐week‐old seedlings of cultivar Attraktion following the protocol from Dvorak *et al*. (1988). Briefly, nuclei were extracted from ground leaves in a sucrose‐based homogenization buffer. The protein contamination was removed by proteinase‐K treatment and phenol:chloroform extraction. The HMW DNA was then spooled out of the solution during sodium acetate and ethanol precipitation. Size profile of the extracted DNA was checked using Femtopulse system genomic DNA 165 kb kit (Agilent Technologies). Eight HiFi SMRTbell^Ⓡ^ libraries were prepared using the SMRTbell^TM^ Express Template Prep Kit 2.0 according to the manufacturer’s instructions (Pacific Biosciences protocol: PN 101‐853‐100 Version 03, January 2020). In short, the protocol involves fragmenting the HMW DNA to a mean fragment length of 20 kb using Megaruptor 3 (Diagenode Inc. 400 Morris Avenue, Suite 101 Denville, NJ 07834 USA), followed by DNA damage repair, end repair/A‐tailing and adapter ligation. Linear DNA fragments were removed by nuclease treatment of the SMRTbell libraries. Size selection of libraries was carried out using the Sage ELF system and fractions with 15–20 kb mean insert sizes were used for sequencing. Polymerase/insert complex formation and clean‐up were performed using Sequel II^TM^ binding kit 2.0 based on the manufacturer’s instructions. Sequencing was performed on 16 8M SMART cells using sequencing chemistry V2.0 and with a 2‐h pre‐extension and 30‐h movie time setting. CCS reads were obtained with PacBio CCS software (https://github.com/PacificBiosciences/ccs).

Chromosome conformation capture sequencing (Hi‐C) libraries were prepared from 1.5 g of leaf material from one‐week‐old seedlings of cv. “Attraktion” as per the protocol of Padmarasu *et al*. (2019) with a few modifications. The modifications include the use of nuclei isolation protocol (Dvorak *et al*., 1988) and Ampure bead‐based size selection instead of SYBR‐gold agarose gel‐based size selection. The prepared library was quantified using qRT‐PCR using known concentration standards and sequenced on two lanes of a NovaSeq 6000 SP flow‐cell using 200 cycles (2 × 100 bp paired‐end mode).

### Data processing and enrichment efficiency calculation

The adapter and low‐quality bases from raw RenSeq reads were removed using cutadapt v1.16 (Martin, 2011) with a minimum read length of 30 bp after trimming. The quality check for adapter and quality trimming was carried out using FastQC v0.11.7 (https://www.bioinformatics.babraham.ac.uk/projects/fastqc/). Trimmed reads were aligned against two versions of the reference genome assembly of cv. Chinese Spring [RefSeq v1.0 and RefSeq v2.1, The International Wheat Genome Sequencing Consortium (IWGSC) (2018); (Zhu *et al*., 2021)] using BWA‐MEM v0.7.17 (Li, 2013) with default parameters. The output was converted to binary alignment map (BAM) format using SAMtools v1.9 (Li *et al*., 2009) and then the sorting was carried out using NovoSort (V3.06.05). Sequences from the bait library were aligned to the RefSeq v1.0 using BLASTn v2.9.0 program (Altschul *et al*., 1990). Alignments with 95% identity and 70% query coverage were retained and alignments separated by 120 bp or less were merged using bedtools v2.29.2 (Quinlan and Hall, 2010). Finally, the total size of regions ≥100 bp in the reference genome covered by alignments to the baits was calculated and considered as the size of our capture target. The sorted BAM file of each genotype was used to calculate the number of reads mapped on target and on the whole genome using SAMtools. The enrichment factor (EF) was then determined as (N/M)/(T/G), where N is the number of reads mapped on the target, M indicates the total number of mapped reads, T denotes the size of the targeted region and G is the size of the genome.

### De novo RenSeq assembly and NBS‐LRR identification

Only genotypes with at least 1 million reads and an enrichment factor ≥100 were considered. The quality trimmed data from each genotype were assembled *de novo* with the CLC Assembly cell (https://digitalinsights.qiagen.com/products‐overview/discovery‐insights‐portfolio/analysis‐and‐visualization/qiagen‐clc‐assembly‐cell/) using the parameters ‐w = 64 ‐p fb ss 200 900. The contigs from each genotype were annotated with AUGUSTUS v3.3.145 (Stanke *et al*., 2006) using wheat gene models as training datasets, and contigs harbouring complete genes were identified. Amino acid (AA), coding sequence (CDS) and transcript sequence for each complete gene were extracted using getAnnoFasta.pl script from the AUGUSTUS package. AA sequences of gene models were used to predict protein domains using the pfam_scan.pl script from PfamScan (Chojnacki *et al*., 2017), which searches FASTA sequences against the Pfam HMM database (Mistry *et al*., 2020). The script was run with a sequence e‐value cutoff of 10^−5^ and domain e‐value cutoff of 0.2 keeping other parameters to default. Genes containing at least one NB‐ARC (NBS) domain (pfam ID PF00931.23) were considered as NLRs and used for downstream analysis. There is no standard tool available to predict the coiled coil (CC) domain, therefore all NLRs with the “Rx_N” (PF18052) domain, which is predicted as coiled coil were classified as coiled coil (CC). The sequences were further classified as NBS (only NBS domain), NLs (NBS + LRRs), CNs (Rx_N + NBS), CNLs (Rx_N + NBS + LRRs), XN (Integrated domain (ID) + NBS), XNL (ID + NBS + LRR), XRN (ID + Rx + N) and XCNL (ID + Rx_N + NBS + LRR) based on domain composition. A bash script was used to retrieve gene structure information such as gene length, number of exons and introns from GFF files. The CDS sequences of NLRs from each genotype were aligned against RefSeq v1.0 using GMAP (Wu and Watanabe, 2005). The alignments were filtered with 70% query coverage and 95% identity cutoff.

### Clustering and saturation analysis

The AA sequences of all the NLRs identified from all genotypes were clustered using the easy‐linclust workflow from MMseqs2 software suite (Steinegger and Söding, 2017). The program was run with e‐value 1e‐15, ‐‐min‐seq‐id 0.95, ‐‐seq‐id‐mode 2 (longer sequences), ‐‐cluster‐mode 2 (coverage of query), ‐‐kmer‐per‐seq 100, keeping other parameters to default. The saturation analysis was carried out to determine the number of genotypes needed to capture 95% OGs. This was done by making a random selection of the genotypes and counting the number of OGs present in these selections. The analysis was carried out separately for all the genotypes, only plant genetic resources and only using elite lines. The process was repeated 100 times starting with two and ending with a maximum number of genotypes for each category.

### Identification of elite and PGR‐specific OGs

The presence‐absence states of the NLRs from each orthogroup (variable *OG*) were used to classify the OG as specific to elite lines, specific to plant genetic resources (PGR) or common (variable *Type*). Further, the effect of OGs on resistance was predicted using the following linear model:

Phenotype=Type+OG

The OGs with negative effects and *P*‐value < 0.01 were considered as resistant OGs. The number of resistant OGs from respective accessions was counted and the correlation of OG count with disease susceptibility was studied.

### Chromosome scale genome assembly of “Attraktion” cultivar

The HiFi reads were assembled using hifiasm v0.14 (Cheng *et al*., 2021) to generate a primary contig assembly. The pseudomolecule construction was carried out using the TRITEX pipeline (Monat *et al*., 2019). For this, the guide map was constructed by aligning the single‐copy sequences from Julius to the Attraktion contig assembly. The Hi‐C data were then used for chimera breaking and contig ordering to generate pseudomolecules.

Transposable elements (TE) were annotated using a homology‐based approach implemented in RepeatMasker v4.0.8 (Smit *et al*., 2004). A custom library was created by downloading and combining wheat TE sequences from ClariTeRep: https://github.com/jdaron/CLARI‐TE) and 2825 complete plant TE sequences (http://botserv2.uzh.ch/kelldata/trep‐db/downloads/trep‐db_complete_Rel‐16.fasta.gz).

Gene annotation was carried out using AUGUSTUS v3.3.1. Initially, CDS sequences of high confidence (HC) genes from RefSeq v2.1 (Zhu *et al*., 2021) were aligned to the Attraktion assembly using GMAP‐GSNAP and Alignment was filtered with 70% coverage and 95% identity and top hits for each gene were extracted. The outputs of RepeatMasker and GMAP‐GSNAP were combined and a GFF file was created. This GFF file along with wheat gene models served as a training dataset for AUGUSTUS.

The assembly completeness was assessed with 1614 Benchmarking Universal Single Copy Orthologs (BUSCO v5.1.2) (Simao *et al*., 2015) genes from plants using “genome mode”. The assembly quality was also evaluated both as genome and gene levels. For genome‐wide comparison, single‐copy sequences from RefSeq v2.1 were aligned to Attraktion assembly using minimap2 v2.17 (Li, 2018). For gene‐level comparison, the transcript sequences of HC genes from RefSeq v2.1 were aligned against the transcript sequences of Attraktion using LAST (Kiełbasa *et al*., 2011). Alignment filtration and synteny analysis were carried out using MCScan (https://github.com/tanghaibao/jcvi/wiki/MCscan‐%28Python‐version%29).

The structural variations (SVs) were detected using the SyRI pipeline (Goel *et al*., 2019) with default parameters. For this, the Refseqv2.1 assembly was aligned to the Attraktion assembly using unimap, a fork of minimap2 optimized for assembly‐to‐reference comparison. The script “sam2delta.py” from RaGOO (Alonge *et al*., 2019) was used for SAM to mummer‐delta format conversion. The delta file was filtered using the delta‐filter utility from MuMmer v4.0 (Marçais *et al*., 2018) to filter out smaller alignments (2000 bp) and the file was converted to TSV format using the show‐coords utility from MuMmer v4.0. The TSV file served as input for SyRI. The SVs from SyRI were reclassified into presence‐absence variations (PAVs), inversions and translocations as follows: The CPL, DEL, DUP/INVDP (loss) variants and the Attraktion sequences in NOTAL and TDM were converted as Absence SVs (relative to Attraktion). The CPG, INS, DUP/INVDP (gain) variants and the query sequences in NOTAL and TDM were converted as Presence SVs (relative to Attraktion). The INV variants were regarded as inversions while the TRANS and INVTR were both regarded as translocation SVs.

### Phenotypic records and analyses

The experimental setup and quality assessment of yellow rust data were already presented in detail elsewhere (Schulthess *et al*., 2021). Briefly, five yellow rust artificially inoculated experiments plus two experiments relying on natural infections were conducted at five different German locations during harvest years 2019 and 2020. In three out of these seven field experiments, the presence of natural leaf rust infection was also recorded. Further details on these experiments can be found in Table S3. Yellow and leaf rust infection severity was expressed in a 1 (no symptoms) to 9 (severe infection) scoring scale according to the Protocols of the German Federal Plant Variety Office (http://www.bundessortenamt.de/internet30/fileadmin/Files/PDF/Richtlinie_LW2000.pdf). Outlier correction within and heritability within and across leaf rust experiments were assessed in the same way as for yellow rust (Schulthess *et al*., 2021). The best linear unbiased estimations (BLUEs) across experiments of yellow and leaf rust were used as phenotypes for downstream analyses (Table S4).

### Reference‐based GWAS

The alignment records against the Chinese Spring reference (RefSeq v2.1) in BAM format (see above) were used for variant identification. The variant calling was performed using the mpileup and call functions from SAMtools v1.9 and BCFtools (v1.8) (Li, 2011). The software was run with the ‐DV parameter for SAMtools mpileup and minimum read quality (‐q) cutoff of 20. The bi‐allelic SNPs were further filtered with minimum QUAL ≥ 40; minimum read depth for homozygous calls ≥2 and minimum read depth for heterozygous calls ≥4 using a custom AWK script. The commands were run in parallel wherever applicable to reduce computational time using GNU parallel (Tange, 2011).

The variant calling was also performed by aligning adapter and quality trimmed reads from each genotype against the genome assembly of Attraktion. Variant calling and SNP filtration were performed as described above except that minimap2 v.2.17 was used for mapping reads against the Attraktion assembly.

The GWAS for yellow rust and leaf rust was carried out using a univariate linear mixed model from GEMMA (v0.98) software (Zhou and Stephens, 2012) with ‐lmm 4 ‐miss 0.2 ‐maf 0.01 parameters while keeping other parameters to default settings. Relatedness and population structure were accounted for using a kinship matrix in form 2*(1‐RD), where RD is the Rogers’ distances between genotypes computed from 17 840 high‐quality GBS SNPs (Schulthess *et al*., 2021). GWAS was also carried out separately using PGRs and elite accessions to identify novel sources of resistance from PGRs, avoiding over‐correction by the kinship matrix.

For paGWAS, SNPs with more than 20% missing data were scored as presence‐absence variants. The presence‐absence status of genotype calls was converted to reference/alternate allele calls. GWAS with these data were done with GEMMA as described above.

### Reference‐free GWAS

The reference‐free GWAS was carried out using the kmersGWAS pipeline (Voichek and Weigel, 2020). Briefly, 31 bp k‐mers that were supported by at least five reads were extracted using kmctools v3.1.1 (Kokot *et al*., 2017). The *k*‐mers from all the genotypes were combined and a nonredundant *k*‐mer presence‐absence genotype matrix was generated. The pipeline was run with 100 permutations, the 5 million top *k*‐mers and minor allele frequency 0.01, while setting other parameters to default. The significance threshold was determined by selecting 5th top *P*‐value from the 100 top *P*‐values obtained from 100 permutations. To position the *k*‐mers in the genome, they were mapped against the reference assemblies of Chinese Spring and Attraktion and positions of uniquely mapped *k*‐mers were retrieved. The positional information along with p‐values was used for the generation of Manhattan plots using the qqman (Turner, 2018) R package.

The significant *k*‐mers were also aligned to the NBS‐LRR transcript database generated above, and the number of *k*‐mers aligned with 100% identity and 100% coverage to representative transcript sequences of each cluster was counted. The results were manually inspected and the candidate clusters with large proportions of significant *k*‐mers for yellow rust and leaf rust were identified.

### Candidate gene identification

The results from various methods mentioned above were compared and consensus regions for yellow rust and leaf rust resistance were determined. The MCscan (https://github.com/tanghaibao/jcvi/wiki/MCscan‐(Python‐version)) software with default parameters was used to study local synteny between different wheat genome assemblies. The CDS sequences of candidate clusters identified based on the kmerGWAS method were aligned separately to each of the consensus regions identified for yellow rust using GMAP (Wu and Watanabe, 2005) and candidate genes were identified. The AA sequences of the NBS domain of candidate genes were extracted and aligned with the NBS domains of cloned R genes from wheat using MAFFT v7.305 (Katoh *et al*., 2002). The spurious sequences or poorly aligned regions were removed using trimAl v1.2 (Capella‐Gutiérrez *et al*., 2009). The phylogenetic analysis was carried out using IQ‐TREE v1.6.12 (Nguyen *et al*., 2015). The phylogenetic tree was visualized using ggtree (Yu *et al*., 2017).

## Conflict of interest

The authors declare that there are no conflicts of interest.

## Author contributions

JCR, NS and MM designed the research. AWS, PHB and JS produced the phenotypic data. SMK and AH performed the capture sequencing. AWS selected the plant material and analysed the phenotypic data. SMK analysed the molecular data and performed the association analyses. BS and BBHW provided the bait library. SP and AH generated PacBio HiFi and Hi‐C data. SMK and MM constructed the reference assembly of cv. Attraktion. SMK, AWS and MM wrote the paper with input from all co‐authors.

## Supporting information


**Figure S1** Distribution of NBS‐LRR orthogroup size in the hexaploid wheat collection
**Figure S2** Intrachromosomal Hi‐C contact matrices for pseudomolecules of cv. Attraktion.
**Figure S3** Whole‐chromosome alignments of the pseudomolecules of cv. Attraktion to Chinese Spring RefSeq v2.1 assembly (Zhu *et al*. 2021).
**Figure S4** Heatmaps showing the levels of sequence identity between Attraktion and other wheat accessions in 1 Mb bins.
**Figure S5** GWAS using SNPs identified relative to the reference assembly of Chinese Spring (RefSeq v2.1).
**Figure S6** Association scans for leaf rust resistance using different marker systems.

**Table S1** Passport data and ENA accession numbers for 907 winter wheat accessions.
**Table S2** RenSeq assembly statistics.
**Table S3** Experimental setup and data quality assessment of leaf rust data.
**Table S4** BLUEs of yellow and leaf rust used for association analyses.

## Data Availability

RenSeq raw data are available from the European Nucleotide Archive (ENA) under accession PRJEB48219. BioSamples IDs of sequence read sets of each genotype are given in Table S1. Yellow and leaf rust phenotypes used for association scans are given in Table S4. The Attraktion genome assembly and the underlying raw data are available under ENA accession PRJEB48529. Variant matrices are accessible at the European Variation Archive under accession PRJEB52597. RenSeq assemblies and annotations are available from the Plant Genomics & Phenomics Research Data Repository (Arend *et al*., 2016) under https://doi.org/10.5447/ipk/2022/4. The DOI was registered with e!DAL (Arend *et al*., 2014).
