# Supplementary material for: A catalogue of resistance gene homologs and a chromosome‐scale reference sequence support resistance gene mapping in winter wheat
Source: Plant Biotechnol J. 2022 May 30;20(9):1730–42. doi: 10.1111/pbi.13843 (PMC9398310; doi:10.1111/pbi.13843)
Supplement: Supplementary file 1 — Figure S1 Distribution of NBS‐LRR orthogroup size in the hexaploid wheat collection Figure S2 Intrachromosomal Hi‐C contact matrices for pseudomolecules of cv. Attraktion. Figure S3 Whole‐chromosome alignments of the pseudomolecules of cv. Attraktion to Chinese Spring RefSeq v2.1 assembly (Zhu et al. 2021). Figure S4 Heatmaps showing the levels of sequence identity between Attraktion and other wheat accessions in 1 Mb bins. Figure S5 GWAS using SNPs identified relative to the reference assembly of Chinese Spring (RefSeq v2.1). Figure S6 Association scans for leaf rust resistance using different marker systems. [file PBI-20-1730-s001.pptx]

## Slide 1
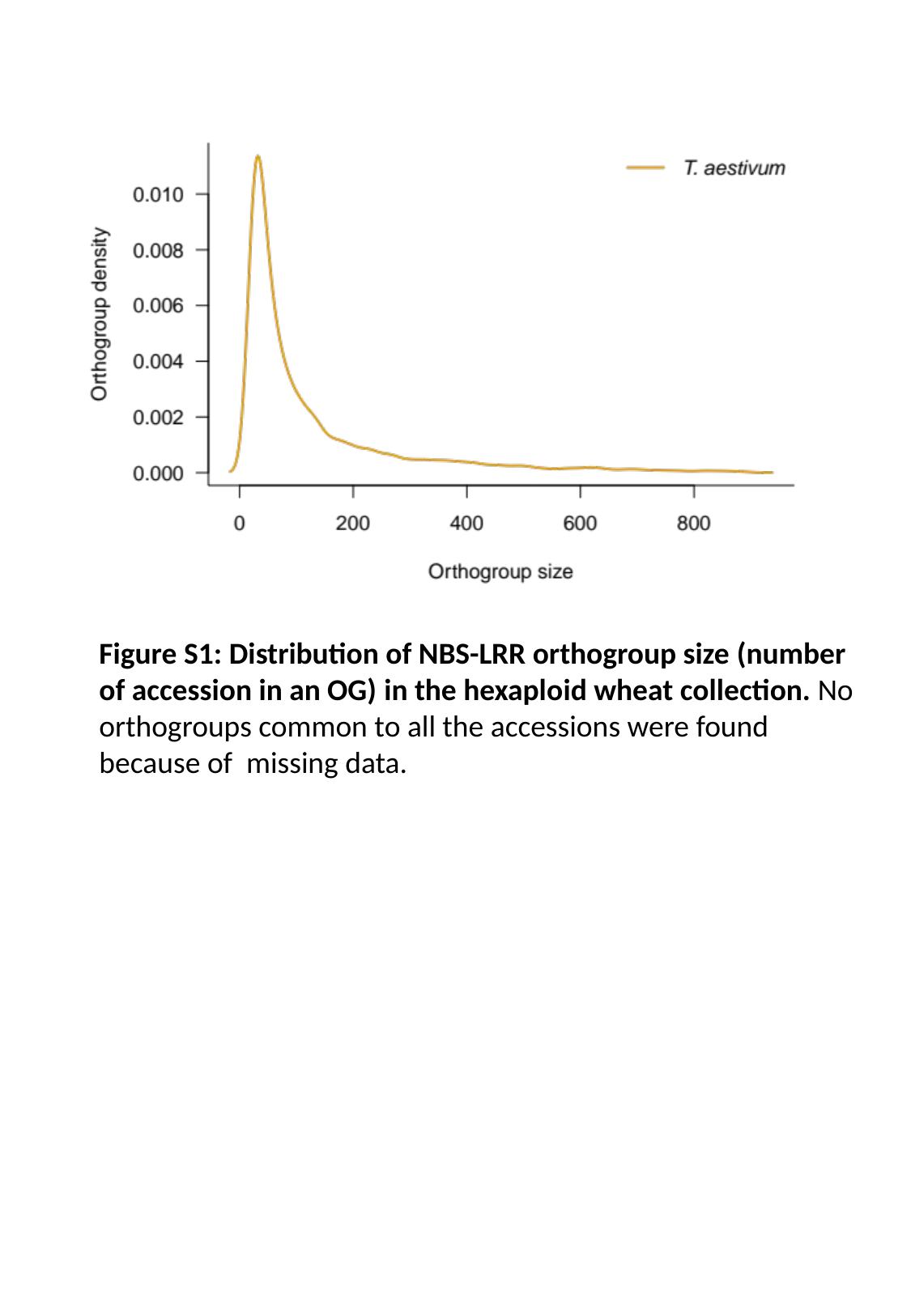

Figure S1: Distribution of NBS-LRR orthogroup size (number of accession in an OG) in the hexaploid wheat collection. No orthogroups common to all the accessions were found because of missing data.

## Slide 2
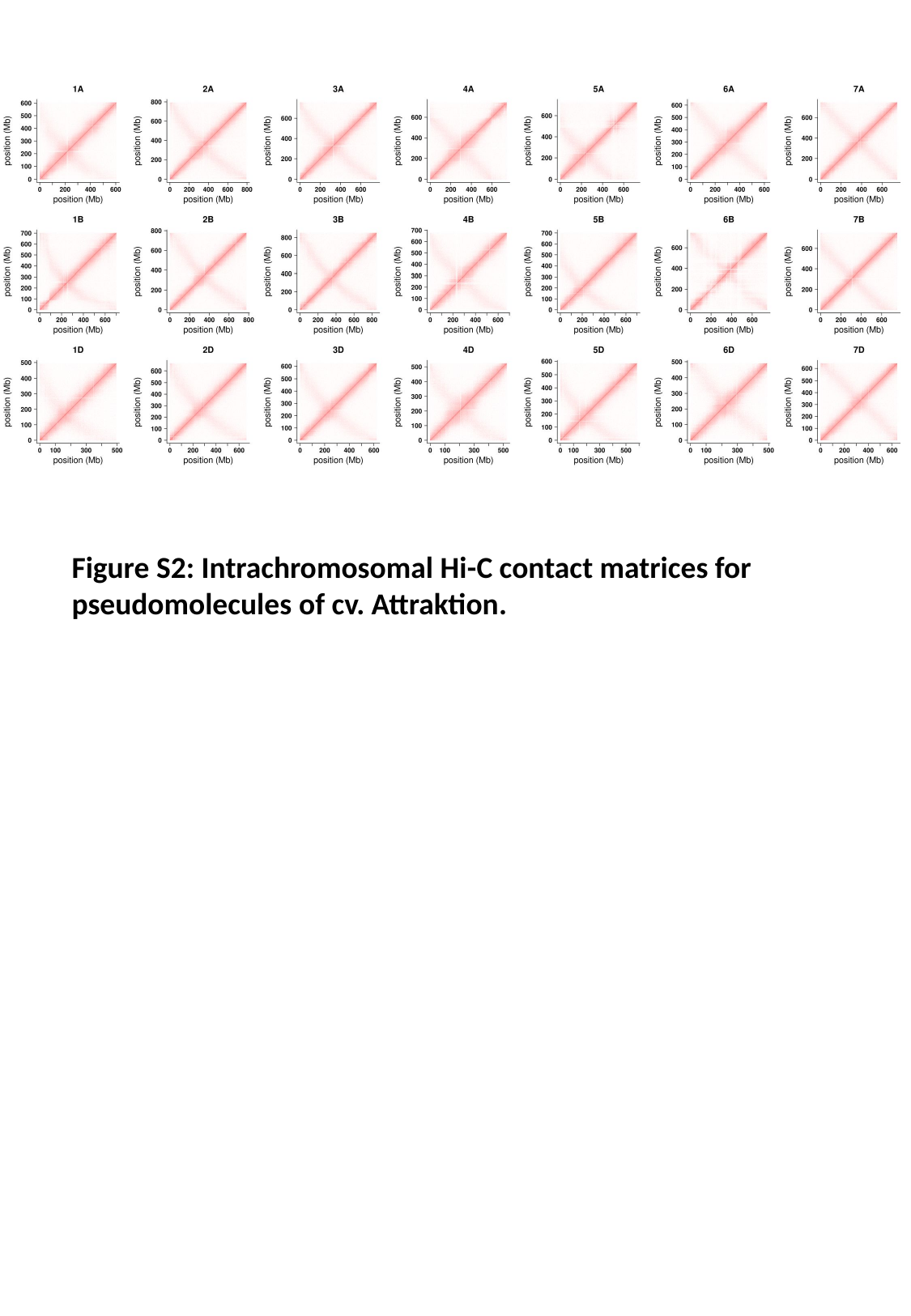

Figure S2: Intrachromosomal Hi-C contact matrices for pseudomolecules of cv. Attraktion.

## Slide 3
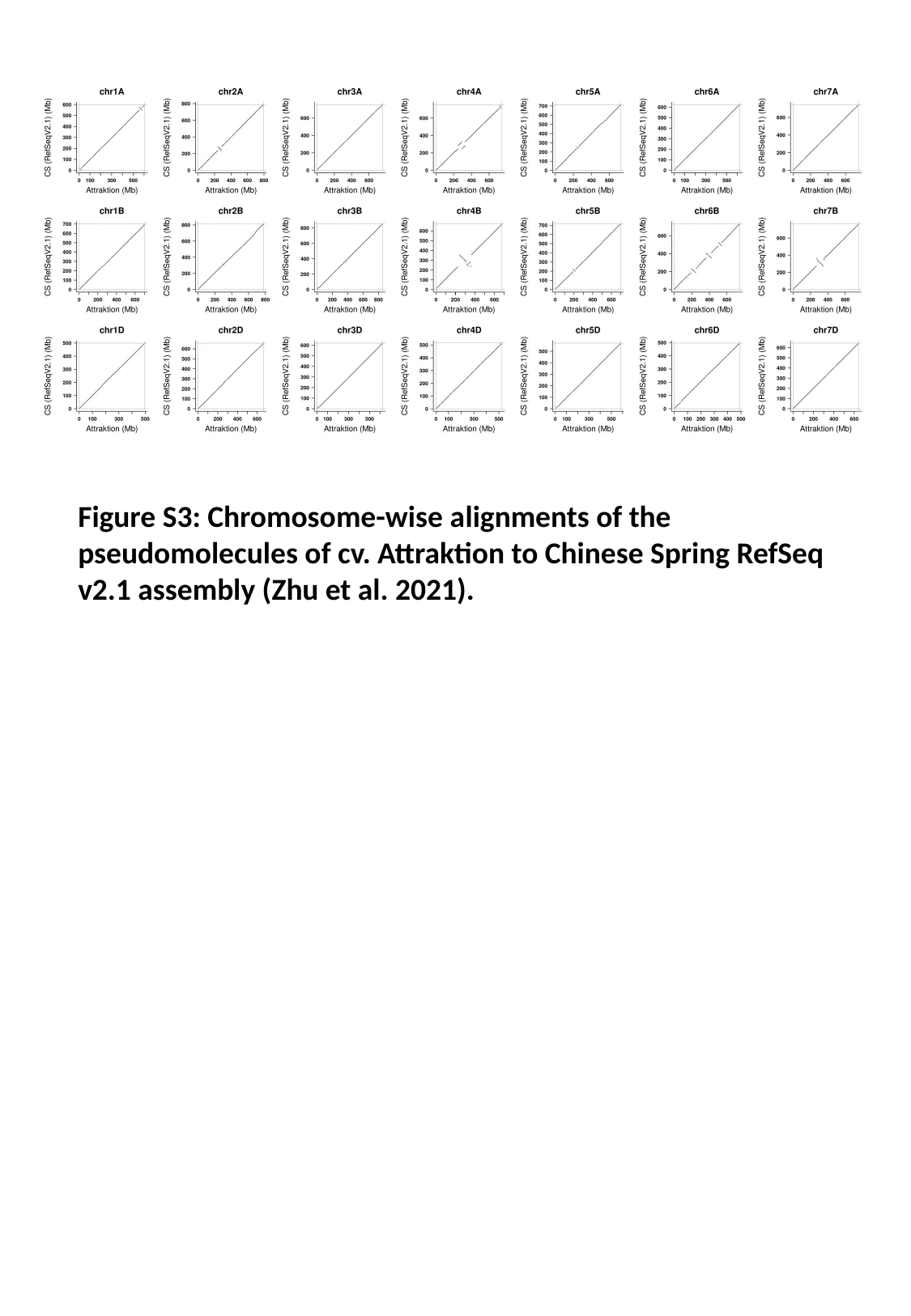

Figure S3: Chromosome-wise alignments of the pseudomolecules of cv. Attraktion to Chinese Spring RefSeq v2.1 assembly (Zhu et al. 2021).

## Slide 4
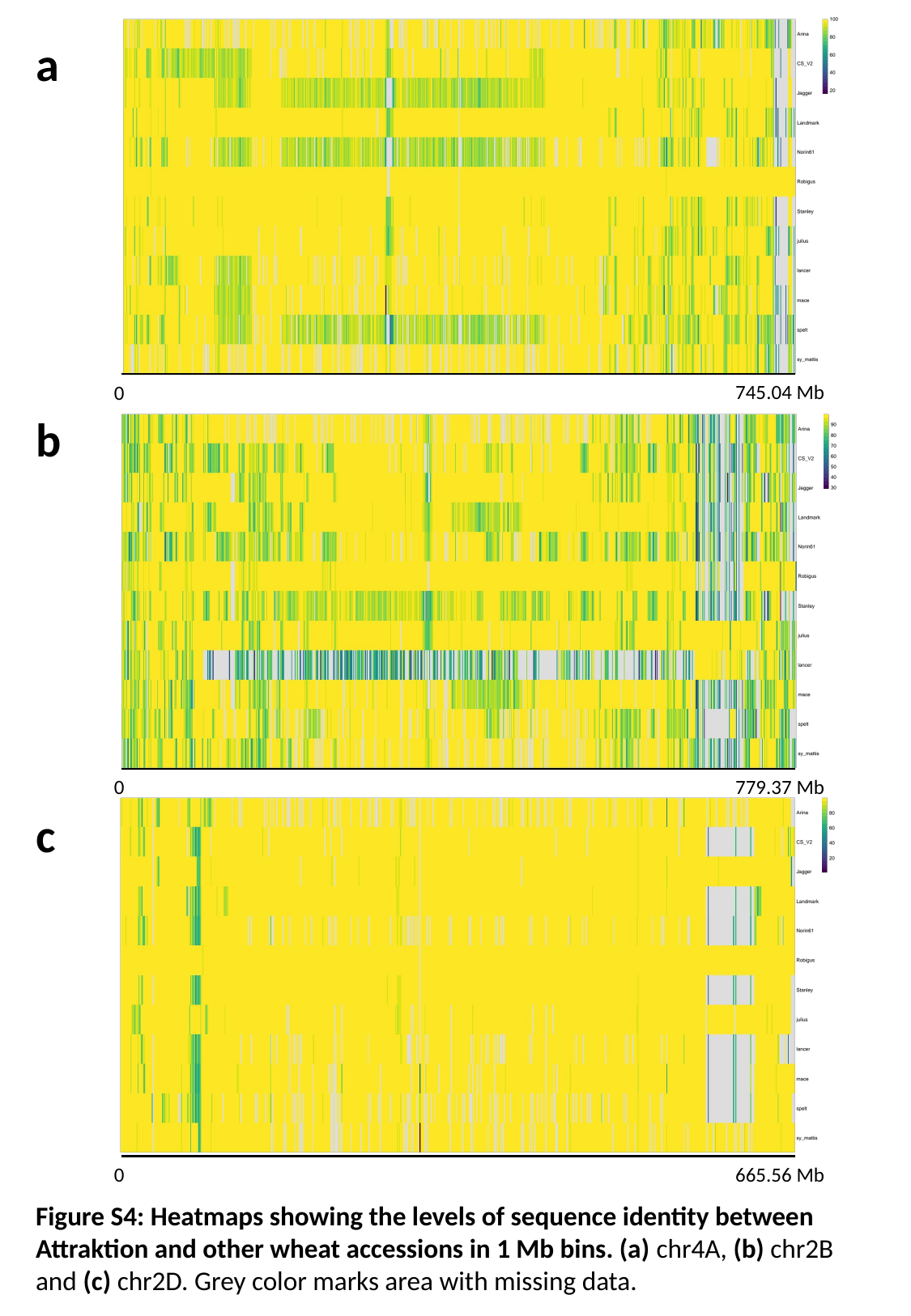

745.04 Mb
0
a
b
779.37 Mb
0
665.56 Mb
0
c
Figure S4: Heatmaps showing the levels of sequence identity between Attraktion and other wheat accessions in 1 Mb bins. (a) chr4A, (b) chr2B and (c) chr2D. Grey color marks area with missing data.

## Slide 5
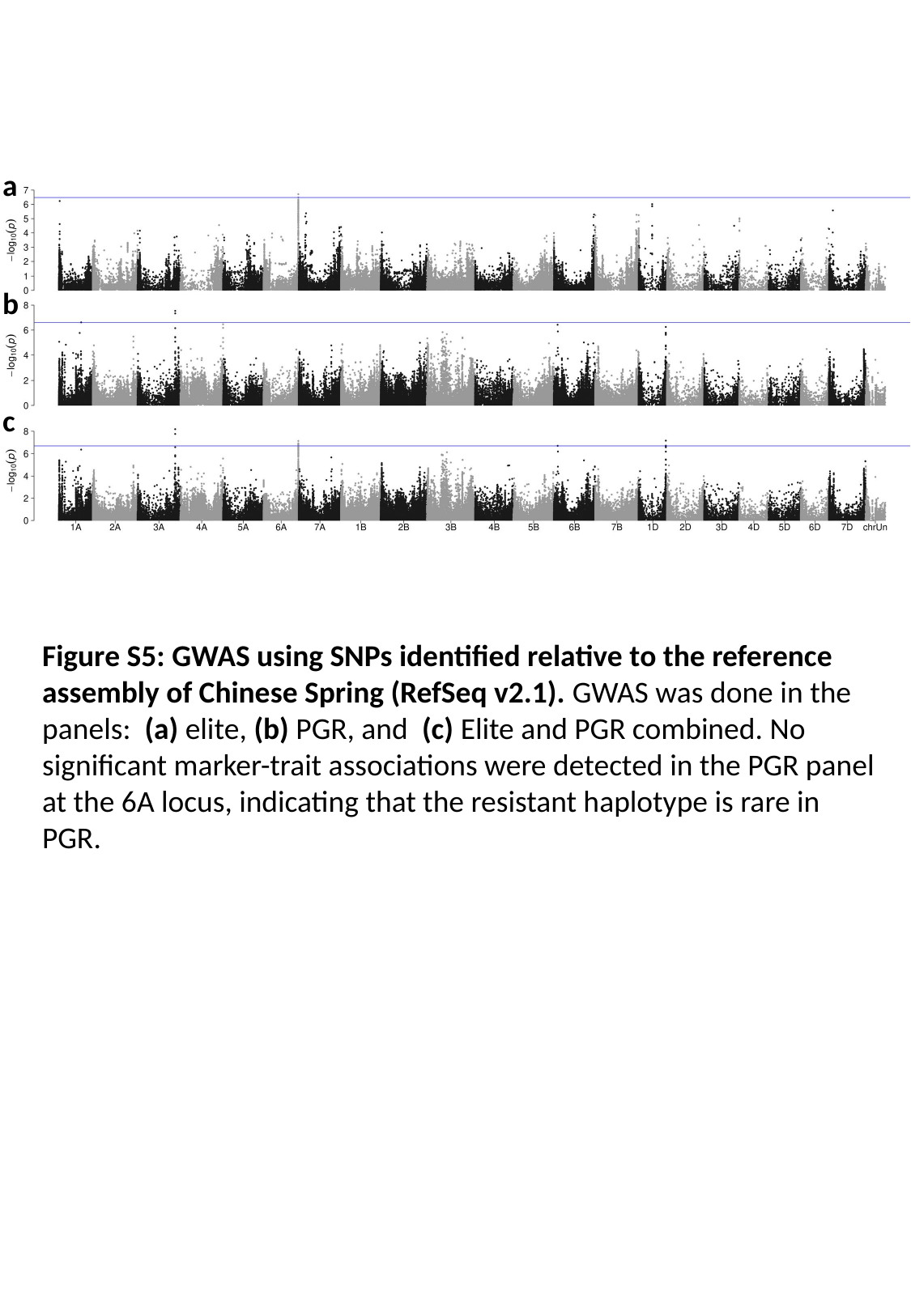

a
b
c
Figure S5: GWAS using SNPs identified relative to the reference assembly of Chinese Spring (RefSeq v2.1). GWAS was done in the panels: (a) elite, (b) PGR, and (c) Elite and PGR combined. No significant marker-trait associations were detected in the PGR panel at the 6A locus, indicating that the resistant haplotype is rare in PGR.

## Slide 6
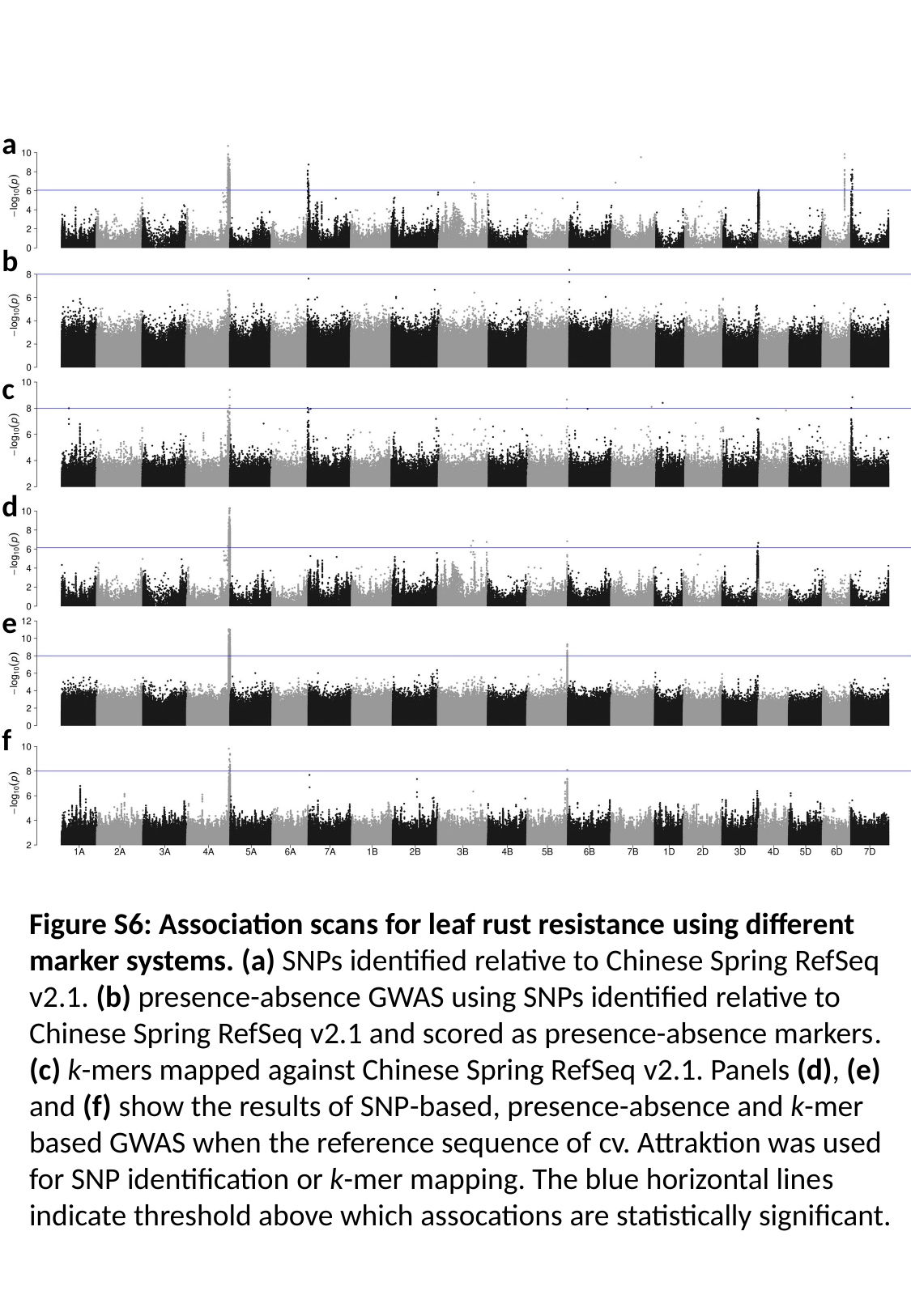

a
b
c
d
e
f
Figure S6: Association scans for leaf rust resistance using different marker systems. (a) SNPs identified relative to Chinese Spring RefSeq v2.1. (b) presence-absence GWAS using SNPs identified relative to Chinese Spring RefSeq v2.1 and scored as presence-absence markers. (c) k-mers mapped against Chinese Spring RefSeq v2.1. Panels (d), (e) and (f) show the results of SNP-based, presence-absence and k-mer based GWAS when the reference sequence of cv. Attraktion was used for SNP identification or k-mer mapping. The blue horizontal lines indicate threshold above which assocations are statistically significant.
